# *Phyllanthus amarus* prevents LPS-mediated BV2 microglial activation via MyD88 and NF-κB signaling pathways

**DOI:** 10.1186/s12906-020-02961-0

**Published:** 2020-07-01

**Authors:** Elysha Nur Ismail, Ibrahim Jantan, Sharmili Vidyadaran, Jamia Azdina Jamal, Norazrina Azmi

**Affiliations:** 1 Drug and Herbal Research Centre, Faculty of Pharmacy, Universiti Kebangsaan Malaysia, Jalan Raja Muda Abdul Aziz, 50300 Kuala Lumpur, Malaysia; 2 Department of Biomedical Sciences, Faculty of Medicine and Health Sciences, Universiti Putra Malaysia, 43400 UPM Serdang, Selangor Malaysia; 3 School of Pharmacy, Faculty of Health and Medical Sciences, Taylor’s University, Lakeside Campus, Jalan Taylor’s, 47500 Subang Jaya, Selangor Malaysia; 4 Immunology Laboratory, Faculty of Medicine and Health Sciences, Universiti Putra Malaysia, 43400 UPM Serdang, Selangor Malaysia

**Keywords:** *Phyllanthus amarus*, Neuroprotection, Microglial activation, Neuroinflammation, BV2 microglial cells

## Abstract

**Background:**

*Phyllanthus amarus* has been shown to attenuate lipopolysaccharide (LPS)-induced peripheral inflammation but similar studies in the central nervous system are scarce. The aim of the present study was to investigate the neuroprotective effects of 80% ethanol extract of *P. amarus* (EPA) in LPS-activated BV2 microglial cells.

**Methods:**

BV2 microglial cells c for 24 h, pre-treated with EPA for 24 h prior to LPS induction for another 24 h. Surface expression of CD11b and CD40 on BV2 cells was analyzed by flow cytometry. ELISA was employed to measure the production of pro-inflammatory mediators i.e. nitric oxide (NO) and tumor necrosis factor (TNF)-α. Western blotting technique was used to determine the expression of inducible nitric oxide synthase (iNOS), myeloid differentiation protein 88 (MYD88), nuclear factor kappa B (NF-κB), caspase-1, and mitogen activated protein kinase (MAPK).

**Results:**

Qualitative and quantitative analyses of the EPA using a validated ultra-high pressure liquid chromatography tandem mass spectrometry (UHPLC-MS/MS) method indicated the presence of phyllanthin, hypophyllanthin, niranthin, ellagic acid, corilagin, gallic acid, phyltetralin, isolintetralin and geraniin. EPA suppressed the production of NO and TNFα in LPS-activated BV2 microglial cells. Moreover, EPA attenuated the expression of MyD88, NF-κB and MAPK (p-P38, p-JNK and p-ERK1/2). It also inhibited the expression of CD11b and CD40. EPA protected against LPS-induced microglial activation via MyD88 and NF-κB signaling in BV2 microglial cells.

**Conclusions:**

EPA demonstrated neuroprotective effects against LPS-induced microglial cells activation through the inhibition of TNFα secretion, iNOS protein expression and subsequent NO production, inhibition of NF-κB and MAPKs mediated by adapter protein MyD88 and inhibition of microglial activation markers CD11b and CD40.

## Background

Neurodegenerative disease such as Parkinson’s disease (PD), Alzheimer’s disease (AD), amyotrophic lateral sclerosis (ALS) and multiple sclerosis (MS) has a common underlying feature: neuroinflammation, characterized by dysregulation in the activation of immune cells of the brain, particularly the microglia [1]. As an active element of the innate immune system, the involvement of microglia has been recognized in these disorders [2, 3]. Microglia activation leads to morphological and functional changes, which result in the transformation from ramified phenotype to amoeboid phenotype, aimed at eliminating foreign bodices [4]. Upon activation, microglia releases different chemokines and cytokines which propagate immune responses [5]. Interaction between microglia and injured neurons produces uncontrolled inflammation and, progresses to brain injury [6]. Anti-inflammatory agents play an increasing role in the treatment of neurological disorders. Non-steroidal anti-inflammatory drugs (NSAIDs) and the classical steroidal anti-inflammatory agents such as dexamethasone are potentially beneficial in the treatment of neurodegenerative diseases with underlying neuroinflammation [7]. Dexamethasone blocks NF-κB activation and suppresses the expression of iNOS, COX-2, TNF-α and IL-1β [8]. However, previous studies have indicated that dexamethasone could also cause CNS injury and could not block CNS cytokine transcription [9].

One of the pattern-recognition receptors (PRRs) is toll-like receptor 4 (TLR4) which is expressed on microglia and involved in the microglia-mediated neuroinflammation. TLR4 is also a central player in orchestrating and initiating both innate and adaptive immune responses [10]. Many studies have demonstrated TLR4-dependent activation of microglia in neurodegenerative disorders [11]. In a healthy adult central nervous system (CNS), microglia communicate with surrounding cells, and keeps the brain environment at normal homeostasis. Lipopolysaccharide (LPS) is a non-infectious component of gram-negative bacterial cells wall. LPS stimulation enables expression of various cell surface molecules on the microglial membrane including the inflammatory biomarkers CD11b and CD40. CD11b and CD40 predominantly express TLR4 which lead to activation of transcription factor, such as nuclear factor-κB (NF-κB) to induce IL-1β, IL-6 and TNFα production [12]. There are two pathways reported for LPS-TLR4-induced signaling: the first is through adapter molecule myeloid differentiation primary-response protein 88 (MyD88) and secondly through MyD88 adapter like (MAL) [13]. The MAPK cascade and its related downstream transcription factor NF-κB were affected after LPS stimulation [14]. These signaling pathways leads to production of inducible nitric oxide synthase (iNOS) and eventually nitric oxide (NO) production, cyclooxygenase-2 (COX-2) and reactive oxygen species (ROS) [15].

*Phyllanthus amarus*, locally known as *dukung anak*, is traditionally used to treat diabetes, jaundice, flu and dropsy [16, 17]. It is reported to have anticancer [18], antioxidant [19], anti-amnesic [20], neuroprotective and neuropathic properties [21]. The major active components of *P. amarus* are phyllanthin and hypophyllanthin [17]. The aqueous extract of *P. amarus* has been used to treat epilepsy and nervous debility [16]. It has been shown to exhibit gastroprotective, antioxidant [22] and hypocholestrolemic activities [23], and could lower blood glucose level [24]. The methanol extract of *P. amarus* has been reported to possess antioxidant activity [25], inhibited phagocytosis [26] and has hepatoprotective properties [27]. The ethanol extract of *P. amarus* (EPA) has been shown to possess various biological properties including anticancer [28], recovery of peripheral nerve after injury [29] and immunosuppression [30, 31]. Studies on anti-inflammatory activity of *P. amarus* within the field of neuroinflammation are currently limited. A recent study focusing on corilagin, an isolated compound from *P. amarus,* demonstrated attenuation of radiation-induced brain injury through microglia activation and the expression of inflammatory cytokines [32]. Other studies using isolated constituents from *P. amarus* were found to partially reverse oxidative damage in stressed rats [33]. There is consistent evidence that stress leads to microglial activity in the hippocampus, and neuroinflammation in particular relates to elevated microglial activity which imply mental illnesses [34]. Some hydrosable tannins (various gallic acid derived esters, gallic acid, derivative of geraniin; phenazine and 3,4,5-thrihydroxybenzoic acid) isolated from *P. amarus* was found to down-regulate protein kinases in rats. Niranthin isolated from *P. amarus* was able to inhibit platelet activation factor-induced paw edema formation in mice [35].

One of the potential therapeutic strategies for neuroinflammation-mediated diseases is through inhibition of microglial surface marker subsets and the TLR4-mediated inflammatory pathway. This reduces the local NO release, proinflammatory cytokine production and subsequently neuroinflammation. The present study was designed to investigate the anti-inflammatory effects of 80% EPA in LPS-stimulated BV2 microglial cells and examined its possible neuroprotective mechanisms.

## Methods

### Chemicals and reagents

Phosphoric acid was kindly provided by Dr. Reezal Ishak (Universiti Kuala Lumpur, Institute of Medical Science Technology, Malaysia). Lipopolysaccharide (LPS; *E.coli* 026:B6) and dimethylsulfoxide (DMSO) was obtained from Sigma-Aldrich, USA. Reference standards including phyllanthin, hypophyllanthin, niranthin, ellagic acid, corilagin, gallic acid, phyltetralin, isolintetralin and geraniin with a purity > 98% respectively used in this research were obtained from ChromaDex (CA, USA). 3-(4,5-dimethylathiazol-2yl)-2,5-diphenyltetrazoleum (MTT), sulphanilamide, *N*-1-naphthylenediamine dihydrochloride, fetal bovine serum (FBS), sodium bicarbonate, accutase, 100 U mL^− 1^ penicillin and 100 μg mL^− 1^ streptomycin, sodium dodecyl sulphate (SDS), skimmed milk powder, Tween-20, β-mercaptoethanol (βME) and bromophenol blue were purchased from Nacalai Tesque Inc., Japan. TNF-α ELISA kit were obtained from R&D System, UK. The antibodies were obtained from Novus Biologicals, USA and Cell Signaling Technology, MA, USA. Nitrocellulose (NC) and, acrylamide and bis-acrylamide solutions, glycerol, Tris-HCL-, Tris base, glycine and methanol were purchased from Bio-Rad, CA, USA. RIPA lysis buffer (10X) and phosphate buffer saline (PBS) tablet was obtained from Merck, Germany. Protease inhibitor cocktail (100X) were purchased from VRW® Life Science Amresco®, USA. Dulbecco’s modified Eagle Medium (DMEM) was obtained from Gibco™, Grand Island, New York, USA. Rat Anti-Mouse CD11b and rat anti-mouse (CD40) was purchased from BD Pharminogen™, BD Bioscience, USA.

### Preparation of hydroethanolic extract of *Phyllanthus amarus*

The preparation of hydroethanolic extract of *P. amarus* was performed as previously described by another group in our laboratory using the same plant sample [36]. *P. amarus* was collected from Marang, Kuala Terengganu, Malaysia in September 2015. The plant was authenticated by Dr. Abdul Latif Mohamad of the Faculty of Science and Technology, Universiti Kebangsaan Malaysia (UKM) and a specimen with voucher number UKMB 30078 was kept at the Herbarium of UKM, Bangi, Malaysia. Plant materials were dried and powdered at room temperature. In the maceration process, 80% ethanol was applied to the powdered plant material for a duration of 72 h. The crude ethanol extract was filtered through Whatmann No. 1 filter paper. The filtrate was evaporated using a rotary evaporator, dried and stored in an airtight container until further use. The extract quantity was calculated by dividing the dried weight of *P. amarus* over the lyophilized EPA. The EPA yield from *P. amarus* was 13.2%.

### Ultra-high performance liquid chromatography analysis

The sample determination was performed on a Perkin Elmer Flexar FX15 UHPLC system coupled to Sciex 3200 hybrid trap triple quad tandem mass spectrometer (UHPLC-MS/MS) using a Phenomenex synergy RP C18, 100A (100 mm × 3 μM × 2.0 mm) column. The UHPLC unit consisted of thermostatted column compartment, vacuum degasser, and binary pump with full loop injection mode. System control and data analysis were carried out by Applied Biosystems Analyst 1.5.2 software. For positive and negative electrospray ionization, a linear binary gradient of deionized water (mobile phase A; with 0.1% formic acid) and acetonitrile (mobile phase B with 0.1% formic acid) were used as mobile phase A and B respectively. Gradient elution started at 95% A (5%B) and changed to 85% B over 8 min and hold for 2 min. Column was then re-equilibrated at 95% A for 2 min. Total run time was 12 min. Flow rate was 400 μL min-1 and the injection volume was kept at 20 μL. The mass spectrometer was operated under positive and negative ionization mode with MRM (multiple reaction monitoring) mode using the following settings: positive polarity mode; source voltage, 5500 V; source temperature, 400 °C; source gas, 40 psi; de-solvation gas, 40 psi; curtain gas, 10 psi. Negative polarity mode; source voltage, 4500 V; source temperature, 400 °C; source gas, 40 psi; de-solvation gas, 40 psi; curtain gas, 10 psi. The data was collected for each sample from a full scan *m/z* 100–1200 and MS/MS scan *m/z* 50–1200.

### Method validation procedures for UHPLC-MS/MS analysis

The precision, linearity, limits of quantification (LOQ) and limits of detection (LOD) was used in the validation process. Intra- and inter-day variation dictated precition. In 1 day, with each dose, three times on three consecutive days the concentration of extracts (1 mg mL^− 1^), and the reference standards (0.0005–0.02 mg mL^− 1^), were injected. The calibration curve was derived from the nine standards of references. A standard curve were constructed by Applied Biosystems Analyst 1.5.2 software from triplicate injections of five concentrations of each standards. The linear calibration analysis measures the linearity and from the calibration curve, correlation coefficient (R^2^) was calculated. The RSD and slope (S) of the calibration were used to measure LOD and LOQ with the following equations: LOD = 3 x (RSD/S) and LOQ = 10 x (RSD/S).

### Cell culture

Mouse BV2 cells were supplied by Dr. Sharmili Vidyadaran (Faculty of Medicine and Health Sciences, Universiti Putra Malaysia). The cells were maintained in DMEM containing 10% fetal bovine serum (FBS), 100 U mL^− 1^ penicillin and 100 μg mL^− 1^ streptomycin. The cells were grown to 70 to 80% confluency before experiments were conducted and maintained at 37 °C in a humidified atmosphere containing 5% CO_2_. The BV2 cells were plated at various densities of 1.25 × 10^4^ cells per mL (96-well plate for MTT assay), 5 × 10^4^ cells per mL (24-well plate for ELISA) and 1 × 10^6^ cells per 800 μl (6-well plate for fluorometric and western blot analysis) and cultured for 24 h. This was followed by treatment with EPA for another 24 h and continued with exposure to LPS (1 μg mL^− 1^) or EPA + LPS for another 24 h. Lyophilized EPA was initially dissolved in 100% DMSO, sonicated and further diluted with PBS to dilute the DMSO to a concentration of 0.1% succeeded by serial dilution for sample preparation. In this study, 0.1% DMSO was used as a vehicle control and dexamethasone phosphate (DEX, 8 μg mL^− 1^) was used as a positive control.

### Cell viability

The BV2 cells (*n* = 3) were exposed to EPA at varying concentrations of 0.5 to 2.5 μg mL^− 1^ in humidified environment overnight. Post incubation, the supernatant was removed for nitrite measurement, and viable cells incubated for another 2 h with MTT (0.5 mg mL^− 1^). Subsequently, the culture medium was removed and the formazan crystals developed were dissolved in DMSO (200 μL). The absorbance was measured at 570 nm (corrected at 600 nm as reference wavelength) using Tecan’s Infinite® 200PRO NanoQuant microplate reader (Tecan Trading AG, Mannedorf, Switzerland). The cell viability was defined as the % untreated control cells [i.e. viability (% control) = 100 x {(absorbance of EPA-treated sample)/(absorbance of control)}].

### Assay of NO production

The level of NO production was observed by measuring the nitrite concentration in the culture medium (supernatant). The BV2 cells were seeded in a 96-well plate (*n* = 3), after 24 h LPS induction, the supernatant was collected. Briefly, equal volume of supernatant and Griess reagent (1% sulphanilamide, 0.1% *N*-1-naphthylenediamine dihydrochloride and 5% phosphoric acid) were mixed for 10 min at room temperature on an orbital shaker. Analysis of NO development was determined at 540 nm using Tecan’s Infinite® 200PRO NanoQuant microplate reader (Tecan Trading AG, Mannedorf, Switzerland).

### Measurement of TNF-α production

BV2 cells were seeded 24-well plate (*n* = 3). The supernatants were collected 24 h after stimulation and the concentrations of TNF-α were measured by enzyme-linked immunosorbent assay (ELISA) using monoclonal antibodies as described by the manufacturer’s protocol (R&D system, MN, USA). The absorbance of the plates was measured at 450 to 570 nm. A standard curve was run on each assay plate using recombinant TNF-α. The kit was specific to TNF-α and did not measure other cytokines.

### Flow cytometric analysis of microglial inflammatory markers

Surface expression of CD11b and CD40 on BV2 cells was analyzed by flow cytometry as described by manufacturer’s instructions. Briefly, BV2 cells were seeded in 6-well plate (*n* = 3), supernatant was removed after 24 h LPS stimulation and cells were washed with warm PBS. All centrifugation steps were carried out at 300 x *g* for 5 min at 4 °C. The cells were centrifuged and washed with ice-cold PBS (twice). One hundred μl of cell suspension (10^6^ cells) were distributed in micro-centrifuge tubes. Next, cells were blocked in a block buffer (1% BSA, 1X PBS, filtered through 0.2 μ syringe filter) and incubated at room temperature for 10 min. Subsequently, CD11b and CD40 and their isotype controls antibodies were added to the tubes respectively, incubated for 20 min on ice and protected from light. The cells were once again washed with ice-cold PBS (twice) and the supernatants were aspirated from the cell pellets with care. After this, the cell pellets were re-suspended with 0.5 mL PBS and kept on ice, protected from light. A minimum of 10,000 events of the gated population is acquired from forward and side scatter plots. Dead cells (debris) are identified based on lower forward and side scatter signal and are eliminated during FACS analysis. Data were analyzed using FACSDiva software v6 (BD FACSCanto II, USA).

### Western blot

BV2 cells were seeded in a 6-well plate (*n* = 3), the supernatant was removed after 24 h LPS stimulation. RIPA buffer supplemented with protease inhibitor cocktail was added to the BV2 cell lysate and homogenized. Twenty μg of total protein was fractionated by SDS-PAGE (12% resolving and 6% stacking) and immunoblotted with phospho-p38 (Thr180/Tyr182), phospho-SAPK/JNK (Thr183/Tyr185), phospho-p44/42 (Erk1/2) (Thr202/Tyr204), NF-κB p65, MyD88, [PVDF membrane; diluted 1:1000 in 5% w/v BSA, 1X TBS, 0.1% Tween-20], iNOS [NC membrane; dilution 1:700 in 5% w/v BSA, 1X TBS, 0.1% Tween-20], β-actin [dilution 1:3000 in 5% w/v BSA, 1X TBS, 0.1% Tween-20], and anti-rabbit IgG, HRP-linked antibody [1:3000 dilution in 5% w/v skim milk, 1X TBS, 0.1% Tween-20]. Immunoblotted protein bands were visualized with enhanced chemiluminescence with WesternBright™ ECL enhanced chemiluminescent substrate kit (Advansta Corporation, San Jose, CA, USA) following manufacturer’s instructions. Quantitative determination of protein expression was performed using Image Lab™ software v5.2.1 (Gel Doc™ XR+, Bio-Rad, CA, USA).

### Statistical analysis

The mean and standard error (SEM) were obtained from a number (*n*) of experiments where *n* = 3. Statistical differences between groups were assessed by one-way analysis of variance (ANOVA) from at least two independent experiments, followed by Dunnett’s test. *p* < 0.05 were considered statistically significant. Graph and statistical analyses were performed using Prism 5 (GraphPad Software, San Diego, CA, USA).

## Results

### Quantitative analysis of hydroethanolic extract of *P. amarus*

**Fig. 1:**
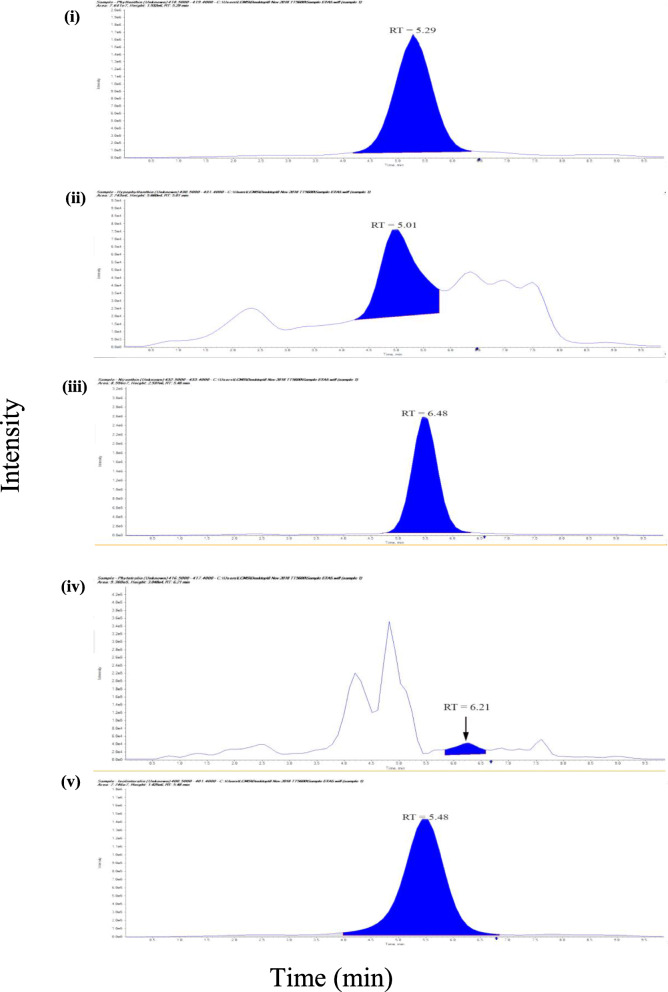
Positive mode: MRM chromatogram of reference standard, lignans. (i) phyllanthin, (ii) hypophyllanthin, (iii) niranthin, (iv) phyltetralin and (v) isolintetralin. RT: retention time

**Fig. 2:**
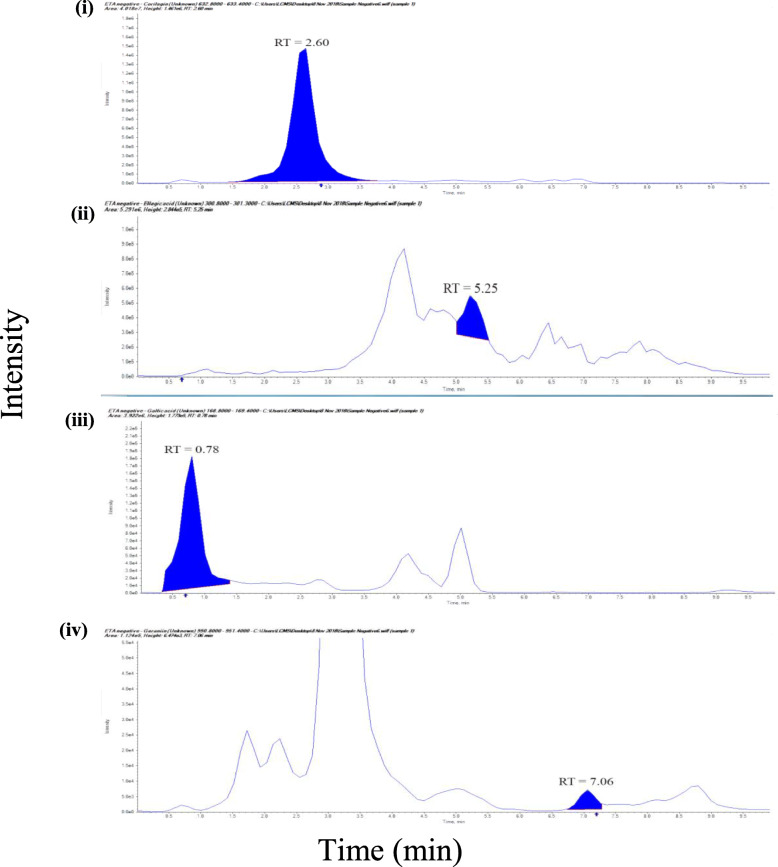
Negative mode MRM chromatogram of reference standard, hydrolysable tannins. (i) corilagin, (ii) ellagic acid, (iii) gallic acid and (iv) geraniin. RT: retention time

The MRM chromatogram of UHPLC-MS/MS of the 80% EPA showed nine compounds (phyllanthin, hypophyllanthin, niranthin, ellagic acid, corilagin, gallic acid, phyltetralin, isolintetralin and geraniin) (Fig. 1 and Fig. 2). Ellagic acid was detected to be the highest at a concentration of 0.116 mg mL^− 1^ followed by corilagin (0.116 ± 0.001 mg mL^− 1^) and niranthin (0.056 ± 0.001 mg mL^− 1^) (*see* Table 1). The peaks were recognized by comparing them with the UHPLC chromatogram of the reference standard of the nine compounds. The calibration curve versus the nine compounds with respective concentration range of 0.0001–0.02 mg mL^− 1^ showed a correlation coefficient (r^2^) of phyllanthin (0.9999), hypophyllanthin (0.999), niranthin (0.997), ellagic acid (0.999), corilagin (0.999), gallic acid (1.000), phyltetralin (0.999), isolintetralin (0.995) and geraniin (0.999) (*see* Table 1). The reproducibility of the results was demonstrated where the RSD (%) values of nine compounds: phyllanthin (6.071), hypophyllanthin (4.417), niranthin (3.412), ellagic acid (4.061), corilagin (4.08), gallic acid (3.512), phyltetralin (4.311), isolintetralin (4.312) and geraniin (2.34) (*see* Table 1). The limit of detection (LOD) and limit of quantification (LOQ) of the nine compounds were phyllanthin (1.0E-6 and 3.0E-6 mg mL^− 1^), hypophyllanthin (1.1E-6 and 3.7E-6 mg mL^− 1^), niranthin (2.0E-6 and 7.0E-6 mg mL^− 1^), ellagic acid (1.5E-6 and 5.-E-5 mg mL^− 1^), corilagin (2.7E-6 and 5.7E-6 mg mL^− 1^), gallic acid (1.2E-6 and 4.0E-5 mg mL^− 1^), phyltetralin (1.0E-6 and 3.0E-5 mg mL^− 1^), isolintetralin (1.0E-6 and 3.0E-5 mg mL^− 1^) and geraniin 1.0E-6 and 3.0E-6 mg mL^− 1^) (*see* Table 1).

**Table 1:** UHPLC-MS/MS quantification analysis of major compounds of *Phyllanthus amarus*

Compound	Concentration (mg mL^**− 1**^)	LOD^**a**^ (mg mL^**− 1**^)	LOQ^**b**^ (mg mL^**− 1**^)	R^**2**^	RSD (%)
Phyllanthin	0.034 ± 0.002	1.0 × 10^− 6^	3.0 × 10^− 6^	0.999	6.071
Hypophyllanthin	0.026 ± 0.001	1.1 × 10^− 6^	3.7 × 10^− 6^	0.999	4.417
Niranthin	0.056 ± 0.001	2.0 × 10^− 6^	7.0 × 10^− 6^	0.997	3.412
Ellagic acid	0.116 ± 0.001	1.5 × 10^− 6^	5.0 × 10^− 5^	0.999	4.061
Corilagin	0.061 ± 0.001	2.7 × 10^− 6^	5.7 × 10^− 6^	0.999	4.08
Gallic acid	0.024 ± 0.001	1.2 × 10^− 6^	4.0 × 10^− 5^	1	3.512
Phyltetralin	< 0.0001 ± 0.129	1.0 × 10^− 6^	3.0 × 10^− 5^	0.999	4.311
Isolintetralin	0.017 ± 0.002	1.0 × 10^− 6^	3.0 × 10^− 5^	0.995	4.312
Geraniin	0.031 ± 0.029	1.0 × 10^− 6^	3.0 × 10^− 6^	0.9990	2.34

^a^Limit of detection, ^b^Limit of quantification

The nine compounds in EPA were identified by comparison with mass fragmentations of the nine reference standards, which were calculated and analyzed by the system software (Applied Biosystems Analyst 1.5.2 software). All targeted compounds are listed in Table 2 with their respective retention times. In positive ion mode, the study identified phyllanthin, hypophyllanthin, niranthin, phyltetralin and isolintetralin (Fig. 1) and, in negative ion mode, ellagic acid, corilagin, gallic acid and geraniin were identified (Fig. 2).

**Table 2:** Retention time and MS/MS fragmentation of nine compounds from *P. amarus*

Compound	Retention time (min)		Molecular ion peak	***m/z*** Fragmentation
	Sample	Reference standards		
Phyllanthin	5.29	5.53	419.5	159
Hypophyllanthin	5.012	5.32	431.5	151, 370
Niranthin	5.484	5.51	433.5	409
Ellagic acid	5.253	5.07	301.2	257
Corilagin	2.601	3.03	633.4	301, 463
Gallic acid	0.782	0.77	169.1	152, 95
Phyltetralin	6.211	6.62	471.5	939
Isolintetralin	5.483	5.82	401.5	377, 393
Geraniin	7.06	7.21	951.4	633, 301

### EPA modulates inflammatory cytokine responses in LPS-activated microglia

**Fig. 3:**
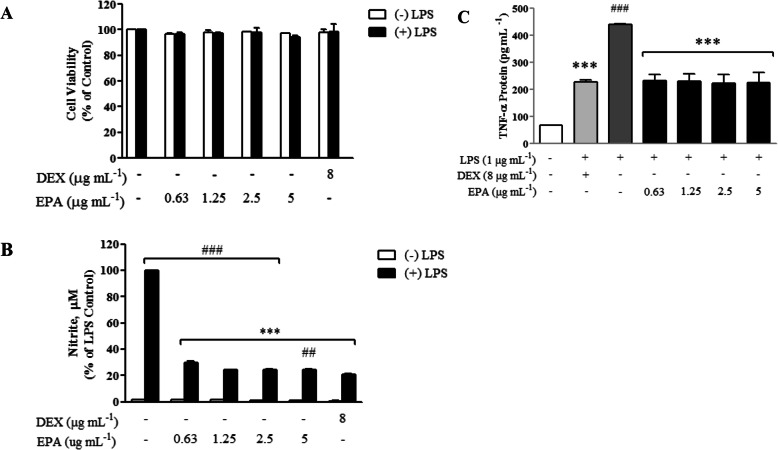
Effects of EPA on cell viability of BV2 cells. Cells were treated with the extract at indicated concentrations or 1 μg ml-1 LPS for 24 h. **a** Cell viability was examined with MTT assay, **b** Nitric oxide analysis with Griess Assay and (**c**) TNFα analysis using ELISA. Results were expressed as the percentage of surviving cells relative to control cells and presented as mean ± SEM of three independent experiments; each was performed in triplicate. Cell viability of treated cells was compared to the respective control sample (cell treated with same amount of vehicle). Statistical differences between groups were assessed by one-way analysis of variance (ANOVA) from at least three independent experiments, followed by Dunnett’s test.* indicate means significantly differ at *p* < 0.05

As a preliminary assessment, the study investigated the potential cellular toxicity of EPA (0.63–5 μg mL^− 1^) on BV2 microglial cells. It was observed in three separate experiments and found that EPA was not toxic to BV2 cells when being treated separately or together with LPS (Fig. 3a, 1 μg mL^− 1^). Clearly, it demonstrated that there was no cytotoxic effect based on MTT cell viability assay (> 95% viable) after 24 h incubation. As detailed in Fig. 3b, NO production was significantly (*p* < 0.05) inhibited by EPA (0.63–5 μg mL^− 1^) in a concentration-dependent manner. The level of TNFα was significantly (*p* < 0.05) decreased to 50% of the levels of LPS as shown in Fig. 3c similar to the level of dexamethasone (DEX).

### EPA inhibits iNOS expression via MyD88 and phospho-kinases leading to alleviation of NF-κB and TNFα levels in BV2 cells

**Fig. 4:**
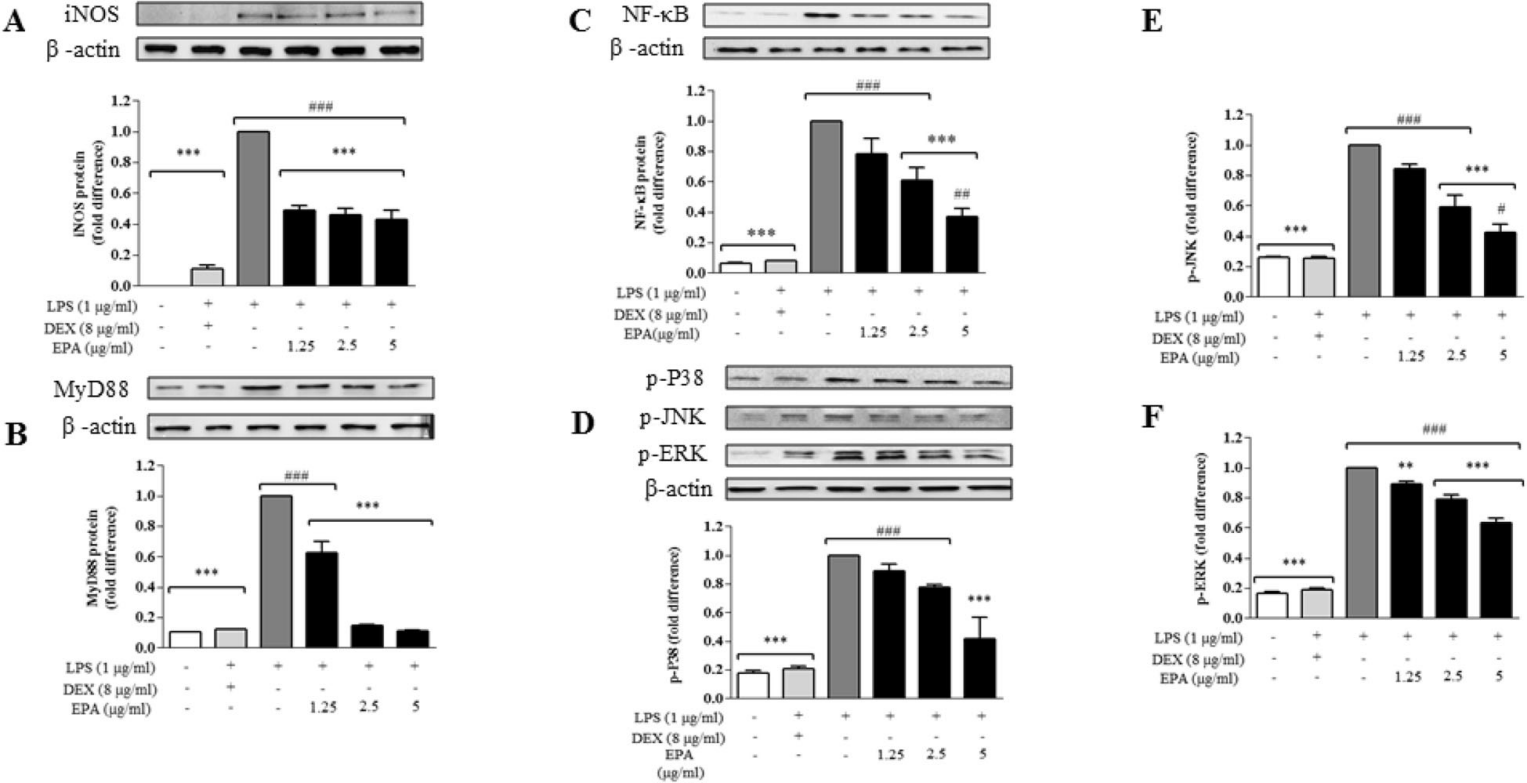
EPA attenuated iNOS via MyD88/MAP kinase pathway, cells were induced with LPS (1 μg ml^− 1^) for 24 h in the presence of EPA. Western blots detecting iNOS (**a**), MyD88 (**b**) NFκB (**c**), (**d**) p-P38, (**e**) p-JNK and (**f**) p-ERK was performed as described in the methodology. Data are shown as mean ± SEM. Statistical differences between groups were assessed by one-way analysis of variance (ANOVA) from at least three independent experiments, followed by Dunnett’s test. * indicates *p* < 0.05 as compared to LPS and # indicates *p* < 0.05 as compared to DEX

EPA has a potential to attenuate iNOS synthesis [37] which synthesizes NO mediated by LPS induction [38]. Figure 4a indicates that although lacking dose-dependency, the levels of iNOS expression and TNFα were significantly (*p* < 0.05) reduced by EPA (1.25–5 μg mL^− 1^) after LPS exposure by 50% or more in BV2 cells. Figure 4b and Fig. 4c show that EPA (1.25–5 μg mL^− 1^) reduced MyD88 signaling and NF-κB after LPS-induction. These results were further confirmed with reduction of phosphor-MAP kinases p-P38 signal at 5 μg mL^− 1^ (Fig. 4d), p-JNK (Fig. 4e) and p-ERK (Fig. 4f) at 2.5 and 5 μg mL^− 1^ (*p* < 0.001). We also investigated the effects of EPA on the pro-inflammatory cytokine IL-1β but detected very low expression of the said cytokine in all treatment groups (data not shown).

### Modulation of inflammatory marker CD11b and CD40 in LPS-induced microglia

**Fig. 5:**
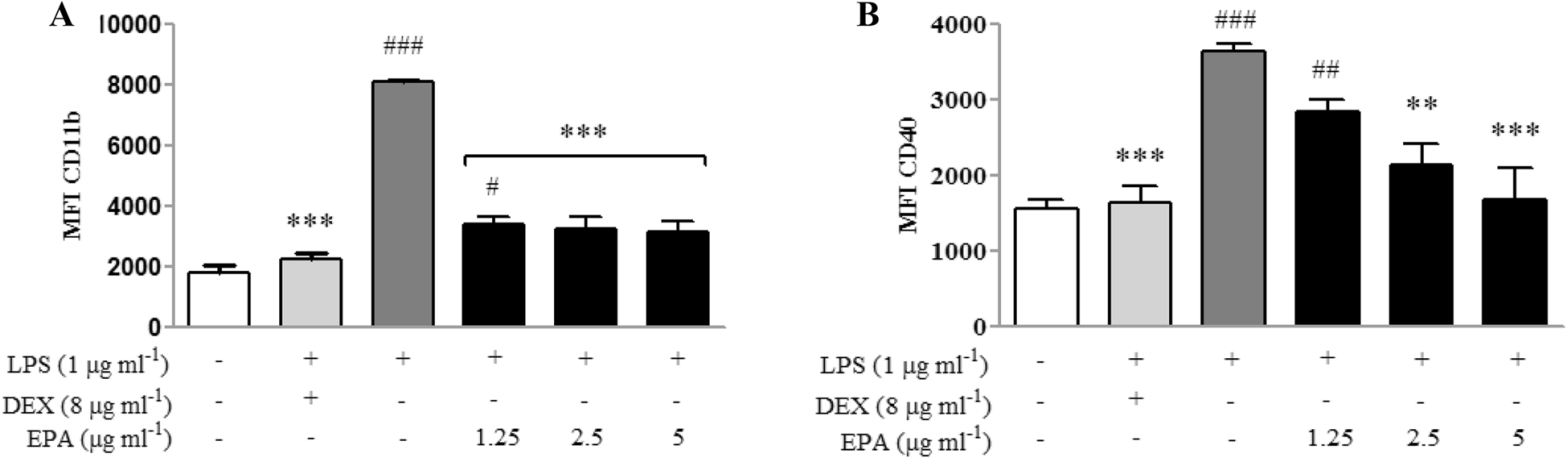
Expression of CD11b (**a**) and CD40 (**b**) on BV2 cells after 24 h LPS-induction prior to a 24 h treatment with EPA or DEX. Statistical analysis of median fluorescence intensity (MFI) of the markers shown in A and B are expressed as mean ± SEM. Statistical differences between groups were assessed by one-way analysis of variance (ANOVA) from at least three independent experiments, followed by Dunnett’s test. * indicates *p* < 0.05 as compared to LPS and # indicates *p* < 0.05 as compared to DEX

Upon LPS stimulation, BV2 adopt an inflammatory phenotype which is characterized by enhanced cell surface expression of key adhesion (CD11b) and costimulatory molecules (CD40). The effect of EPA treatment on BV2 cell surface expression of CD11b and CD40 in basal and LPS-treated BV2 cells is shown in Fig. 5. Flow cytometric analysis (FACS) of marker median fluorescence intensity (MFI) following stimulation with 1 μg mL^− 1^ LPS CD11b (Fig. 5a) and CD40 (Fig. 5b) was significantly (*p* < 0.05) escalated and these changes in expression were inhibited in the presence of EPA (1.25–5 μg mL^− 1^) for CD11b. CD40 (5 μg mL^− 1^) appeared to be the most potently regulated marker that completely inhibited LPS-induced upregulation.

## Discussion

It was shown that EPA has diverse biological properties including hepatoprotective [27], phagocytic inhibition [26], anti-inflammatory [36, 37] and anti-tumor activities [18]. However, there is limited evidence on the effects of EPA in neuroinflammation. To our knowledge this is the first report indicating that EPA inhibits LPS-induced inflammatory responses and attenuates the activation of CD11b and CD40 in microglial cells.

In this study, UHPLC-MS/MS was used for qualitative profiling of EPA against nine standard of reference: phyllanthin, hypophyllanthin, niranthin, ellagic acid, corilagin, gallic acid, phyltetralin, isolintetralin and geraniin. Our UHPLC-MS/MS assays were linear of at least from 0.0005–0.02 mg mL^− 1^ range of concentrations. Previous studies using LC-MS/MS included both qualitative and quantitative analysis of the aerial part of *P. amarus* on two major bioactive compounds namely phyllanthin and hypophyllanthin [39, 40]. A similar linear range to our phyllanthin, hypophyllanthin and gallic acid compounds was reported with higher concentrations LOD and LOQ [40, 41].

In previous studies, several phytoconstituents have been identified and isolated from *P. amarus* such as tannins, flavonoids, tetracyclic triterpenoids and polyphenolic compounds [17, 42], lignans and hydrolysable tannins [18, 43] which include nine of the reference compounds used presently. These compounds might be responsible for the observed anti-inflammatory effects mediated by changes in the signaling pathway similar to those previously reported [26, 31, 38]. Our preliminary study has shown that three out of these nine compounds; namely phyllanthin, ellagic acid and gallic acid, have demonstrated neuroprotective activities against LPS-induced activation of BV2 microglial cells when pretreated with these compounds individually (data not shown).

Immortalized BV2 microglial cell line is used in research related to neurodegenerative disorders because they have similar activation markers, phagocytic function releasable factors and motility to those of primary microglia. CD11b are molecular markers for microglia identification [41] and CD40 (a costimulatory molecule) are expressed by microglia upon activation [44]. CD11b and CD40 participate in pathologic processes in various human diseases, especially inflammatory and autoimmune diseases [44, 45]. According to Zhang et al. (2013) CD11b is upregulated with subsequent LPS-induced microglial activation. Harun and colleagues has reported that CD40 (a member of tumor necrosis factor receptor (TNF-R) superfamily), expressed on the cell surface of microglia, triggers a series of intracellular signaling events which subsequently propagate inflammation after LPS induction [44]. In this study, we found that LPS stimulation significantly (*p* < 0.05) increased CD11b and CD40 expression. Pre-treatment with EPA inhibited this effect in a concentration-dependent manner. A previous study indicated that activated microglial cells also expressed other biomarkers such as CD11c, CD68 and LN-3 [44]. Therefore, it is worth to explore the effects of EPA on these markers.

Chronic activation of microglia gives rise to various neurotoxic mediators and pro-inflammatory cytokines which may lead to neurodegeneration progressing to neurodegenerative disorders [46]. The present study was designed to determine the effects of EPA in LPS-induced BV2 microglial cells. Our present findings showed that NO production was inhibited and iNOS protein expression was suppressed. The secretion of TNFα in LPS-activated murine microglial cells was also decreased. Various research evidences indicated that upregulation of iNOS leads to higher NO production [47] resulting in neurotoxic effects and is associated with several neurodegenerative disorders [48–50]. In addition, local release of TNFα amplifies the inflammatory reaction in the brain by recruitment of peripheral macrophages across the blood-brain barrier, sequentially leading to neuroinflammation [51]. These findings support the notion that EPA reduced neurotoxic mediators (NO) and pro-inflammatory cytokine (TNFα) during inflammation and may be able to reduce LPS-induced inflammatory responses in the brain. Furthermore, EPA showed a significant (*p* < 0.05) inhibition of iNOS protein expression which resulted in a decreased NO production [52, 53] suggestive of a neuroprotective action. Our laboratory has demonstrated the in vivo anti-inflammatory effects of pre-treatment with ethanolic extract of whole plant on LPS-induced neuroinflammation [30, 54] and peripheral inflammation [31]. Indeed, we have also demonstrated neuroprotective effects of EPA against LPS-induced memory impairment in rodents [30]. Therefore, the present study sought to investigate the protective effect of the same ethanolic extract on the nervous system using microglial cells to elucidate the underlying cellular mechanisms of its protective actions. In the present study, very low expressions of IL-1β were detected after pre-treatment with EPA in LPS-induced BV2 microglial cells. Recent studies found similar findings not only in BV2 cells but also in other cell lines [55–57]. In addition, Bussi and colleagues were able to identify TNFα and IL-6 in microglial cells [56]. However, the present study did not look at the involvement of IL-6, another important pro-inflammatory cytokine, which warrants further investigations.

Toll-like receptors (TLRs) are the key cell-surface architect of inflammatory responses to pathogens [58]. Upon stimulation of TLR4 particularly, by ligands such as LPS, adapter protein MyD88 is employed as a signal machinery to form TLR/MyD88 complex [47]. MyD88 acts as a link between receptors and downstream signaling molecules, thus an adapter protein for vast majority of TLRs. This study evaluated EPA effects on MyD88/NF-κB/MAPKs protein expression after LPS-activation in BV2 cells. Overexpression of TLR4/MyD88 will induce the release of pro-inflammatory mediators and cytokines cascade [59] by activating downstream kinases leading to activation of NF-κB and its downstream transcription factor MAPK cascade [60, 61]. Presently, we observed that EPA attenuated MyD88 signaling in LPS-activated microglia. Upon observing further downstream signaling, EPA markedly decreased phosphorylation of MAPK such as P38, ERK and JNK induced by LPS. In addition, EPA also suppressed the activation of NF-κB. Taken together, these results suggest that EPA inhibited the release of pro-inflammatory cytokines and mediators of inflammation through inhibition of TLR4/MyD88/ NF-κB/MAPKs signaling pathways in the microglia upon stimulation by LPS. Although EPA inhibited iNOS and TNFα similarly at all concentrations, further investigations looking into the gene expression level are required to further explain the lack of dose-dependent effects on expression of these proteins.

Dexamethasone is a potent immunosuppressant that inhibits the cytokine production induced by bacterial lipopolysaccharides [62]. Therefore, it was able to reduce the expression of a majority of marker proteins near to the control levels in the treated cultures. It was evident from our findings that dexamethasone was more effective than EPA itself. Although the phytocompounds found in EPA could exert the same effects when exposed to the cells individually, the concentrations used for EPA in the present study contained a combination of these compounds and many others at very low concentrations. The effects of EPA on LPS-induced inflammation were still significant (*p* < 0.05) although lower than dexamethasone. Similar effects were also observed in many studies using different models of inflammation [26, 36, 38]. Taken together, it becomes increasingly evident that EPA has the potential to be developed as a natural product for the prevention of inflammation, not as a drug per se.

The present study indicates a role for EPA in modulating anti-inflammatory responses in BV2 microglial cells with a subsequent neuroprotective action. Therefore, the inhibition of microglial activation is potentially therapeutic in preventing neuroinflammation and plausibly the progression of neurodegenerative diseases. For future studies we suggest to look into the combination effect between EPA and dexamethasone to investigate the neuroprotection action in LPS- induced the activation of microglial cells.

## Conclusions

**Fig. 6:**
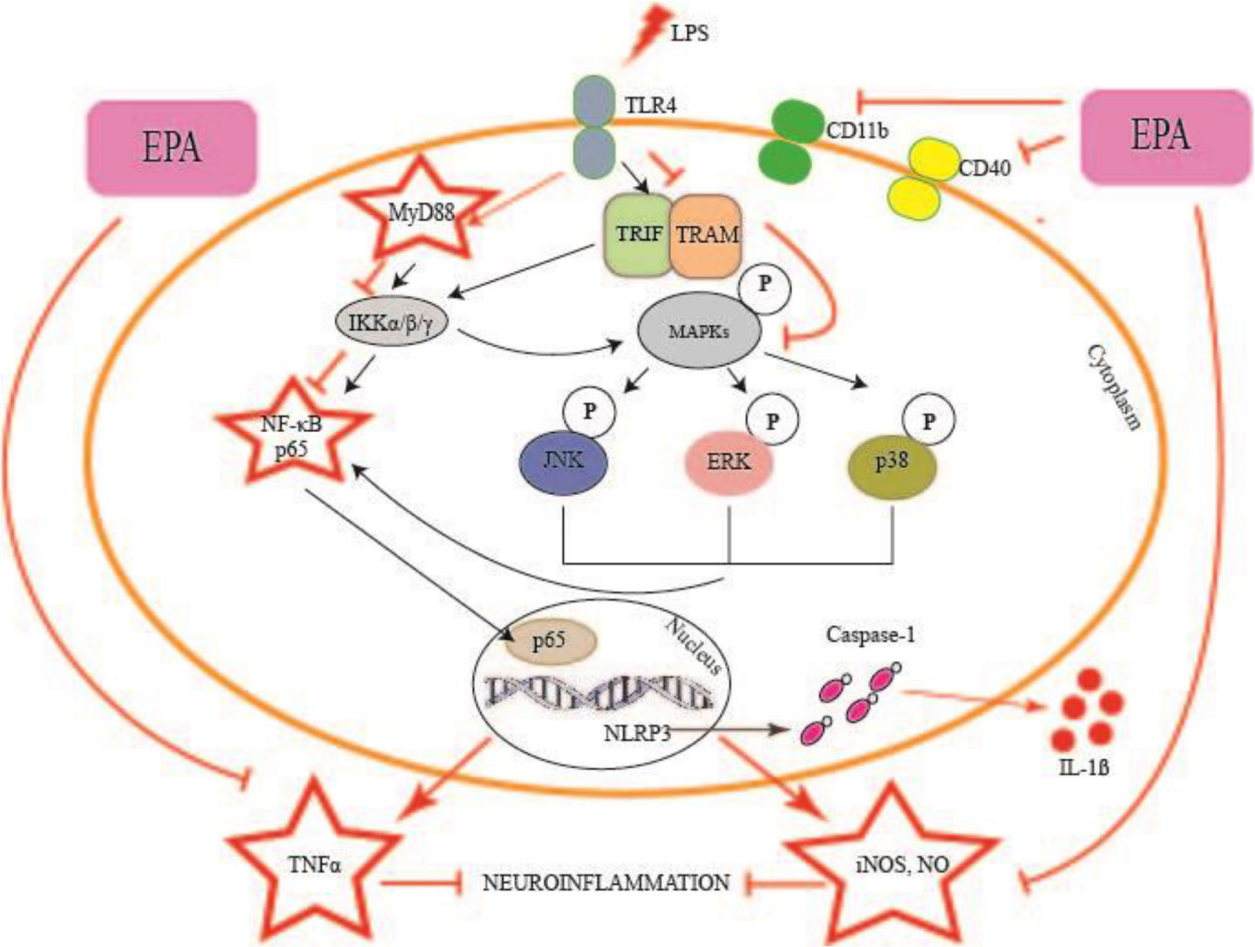
The proposed neuroprotective effects of EPA in BV2 microglial cells challenged with LPS through inhibition of the processes involved in neuroinflammation. EPA: ethanolic *P. amarus* extract, LPS: lipopolysaccharide, TLR4: toll-like receptor 4, TRAF6: THF receptor associated factor 6, IKK: IκB kinase, MAPK: mitogen activated protein kinase, JNK: c-Jun N-terminal kinase, ERK: extracellular-signal-regulated kinase, P38: p38 mitogen-activated protein kinase, NF-κB:nuclear factor kappa light chain enhancer of activated B cells, TNFα: tumor necrosis factor alpha, IL: interleukin (e.g., IL-1β, IL-6), iNOS: inducible nitric oxide synthase, PGE2: prostaglandin E2, COX-2: cyclooxygenase-2

Figure 6 summarizes our findings and the proposed mechanisms of EPA neuroprotective actions against LPS-induced microglial cell activation through inhibition of TNFα secretion, iNOS protein expression and subsequent NO production, inhibition of NF-κB and MAPKs mediated by adapter protein MyD88 and inhibition of microglial activation markers CD11b and CD40. Future work should aim to further elucidate the mechanisms of EPA protective actions through alteration of TLR4 signaling pathway and mRNA expression of proinflammatory factors.

## Data Availability

The datasets generated and/or analyzed during the present study are available from the corresponding author on reasonable request.

## Abbreviations

CD11b: Cluster of difference 11b
CD40: Cluster of difference 40
ELISA: Enzyme-linked immunosorbent assay
EPA: *P. amarus* Ethanolic extract
iNOS: Inducible nitric oxide synthase
LPS: Lipopolysaccharide
MAPK: Mitogen activated protein kinase
MyD88: Myeloid differentiation protein 88
NF-κB: Nuclear factor kappa B
NO: Nitric oxide
*P. amarus*: *Phyllanthus amarus*
TNFα: Tumor necrosis factor alpha
UHPLC-MS/MS: Ultra high pressure liquid chromatography tandem mass spectrometry
